# Drug-related problems in hospitalized patients with chronic kidney diseases and clinical pharmacist interventions

**DOI:** 10.1186/s12877-023-04557-y

**Published:** 2023-12-13

**Authors:** Su Zhang, Guo-bing Zhang, Ping Huang, Yan Ren, Bo Lin, Yan-fei Shao, Xiao-lan Ye

**Affiliations:** 1 Center for Clinical Pharmacy, Cancer Center, Department of Pharmacy, Zhejiang Provincial People’s Hospital(Affiliated People’s Hospital), Hangzhou Medical College, Hangzhou, Zhejiang China; 2 Urology & Nephrology Center, Department of Nephrology, Zhejiang Provincial People’s Hospital (Affiliated People’s Hospital), Hangzhou Medical College, Hangzhou, Zhejiang China

**Keywords:** Drug-related problems, Chronic Kidney Disease, Contributing factor, Clinical pharmacist

## Abstract

**Background:**

Patients with chronic kidney disease (CKD) are at high risk of drug-related problems (DRPs) because of extensive comorbidities and pharmacokinetic changes. This study aimed to identify DRPs and possible contributing factors in hospitalized patients with CKD, and evaluate the efficacy of the clinical pharmacist services in detection and intervention of DRPs in a large general hospital in Zhejiang Province, eastern China.

**Methods:**

With the approval of the Ethics Committee, patients with CKD admitted to the nephrology ward from January to December 2020 were enrolled in this prospective study. The clinical pharmacist identified and intervened the DRPs during hospitalization. The DRPs were classified using the Pharmaceutical Care Network Europe (PCNE) DRP classification system, and all data were statistically analyzed using Statistical Package for Social Science (SPSS) version 26.0.

**Results:**

A total of 914 patients with CKD were included, with 463 DRPs observed among 420 (45.95%) participants; the average DRP per patient was 0.51 (standard deviation [SD], 0.60) before pharmacist intervention. Treatment safety accounted for the highest proportion of problems (43.84%), followed by treatment efficacy, accounting for 43.20%. Drug selection was the most common cause of DRPs (60.26%), and antibiotics and cardiovascular agents were the most common drugs associated with DRPs (32.84% and 28.66%, respectively). A total of 85.53% of pharmaceutical intervention recommendations were followed, and 84.23% of DRPs were completely resolved after intervention by the clinical pharmacist. The proportion of patients who experienced DRPs decreased to 7.77%, with an average of 0.08 (SD 0.28) DRPs during hospitalization after pharmacist’s intervention. Significant contributing factors for DRPs were CKD stage 4, number of comorbid diseases, number of prescribed medications, and hospitalization days in both the univariate and multivariate logistic regression models.

**Conclusion:**

DRPs are common among hospitalized patients with CKD in China. CKD stage 4, the number of comorbidities, use of multiple prescription drugs, and extended length of hospital stay are contributing factors for DRPs. Even only one clinical nephrology pharmacist in the nephrology ward, clinical pharmacist can play an important role in facilitating the identification of DRPs in patients with CKD and assisting physicians resolve DRPs in this single center study in China.

**Supplementary Information:**

The online version contains supplementary material available at 10.1186/s12877-023-04557-y.

## Background

In 2017, the global prevalence of chronic kidney disease (CKD) was estimated to be 9.1%, with 697.5 million cases of all-stage CKD recorded [1]. China accounted for 132.3 million among these cases with the incidence was 9.57% [1]. A 2014 cross-sectional study estimated the prevalence of CKD at 9.88% (approximately 5.67 million patients) in Zhejiang Province, eastern China [2]. According to the 2015 Annual Data Report of the China Kidney Disease Network (CK-NET), more than half of patients with CKD in China were aged ≥ 60 years, and the most common causes of CKD include diabetic kidney disease, hypertensive nephropathy, obstructive nephropathy and glomerulonephritis [3].

According to the Pharmaceutical Care Network Europe (PCNE), drug-related problems (DRPs), including unnecessary drug therapy, ineffective drugs, need for additional drug therapy, and inappropriate drug dose or frequency, can certainly or potentially affect the desired therapeutic outcomes [4–6]. A systematic review in 2020 based on 16 studies reported that the average number of DRPs per patient ranged from 0.58 to 7.2 [7]. A large population-based retrospective study found 25% of patients with CKD had three or more comorbidities, with hypertension, diabetes, heart failure, chronic pulmonary disease and atrial fibrillation being the five most common comorbidities [8]. The use of multiple drugs is necessary in CKD population with comorbidities, but also obviously increase the risk of DRPs [5, 9–11]. Besides, CKD seriously alters the pharmacokinetic parameters of drugs mainly metabolized and excreted by kidney [10, 12]. Some antibiotics, such as vancomycin, need to adjust the dose or frequency according to kidney function in CKD population. The dose-adjustment regimens might be more complex, especially in the CKD cases with renal replacement therapy [12, 13]. Briefly, due to extensive comorbidities and pharmacokinetic changes, the patients with CKD are at high risk of DRPs [5, 14]. A systematic review based on 20 studies showed that the prevalence of DRPs in cases of CKD ranged from 12–87% [14].

Since DRPs are highly prevalent in patients with CKD, it is necessary to discover and address DRPs in time. The valuable contribution of pharmacists to drug therapy in patients with CKD, including drug dosage adjustment, adverse reaction detection, blood concentration monitoring, and medication-related education, has been documented in two systematic reviews [15, 16]. It can be predicted that with pharmacist identification and intervention of DRPs, the drug use in patients with CKD will be more reasonable and clinical outcomes will be greatly improved [16–21]. In a 2-year randomized, controlled study, compared with patients receiving the standard of care, those receiving pharmaceutical care had significantly fewer all-cause hospitalizations and shorter lengths of stay; 530 DRPs were simultaneously identified and resolved [22]. In a randomized controlled trial including 100 patients with CKD, the number of DRPs per patient at discharge was significantly affected by clinical pharmacists, and was 0.94 ± 1.03 and 1.96 ± 1.25 in the intervention and control groups respectively (p < 0.001) [20].In the Zhejiang Province, there are approximately 5.67 million CKD patients and only approximately 15 nephrology pharmacists [2]. It is common for many public hospitals in Zhejiang Province, even in China, to have only one clinical nephrology pharmacist, under the background of relatively insufficient number of clinical nephrology pharmacists. So far, the characteristics of DRP in Chinese patients with CKD has not been described in the literature. The aim of the present study was to identify DRPs and possible determinants in hospitalized patients with CKD, and evaluate the efficacy of the clinical pharmacist services in detection and intervention of DRPs in a nephrology ward of a large general hospital in Zhejiang Province, eastern China.

## Methods

### Study design and setting

This prospective study was approved by Ethics Committee of Zhejiang Provincial People’s Hospital. Hospitalized patients admitted in the nephrology department (60 beds) with diagnoses including CKD from January to December 2020, were included in this study after providing informed consent. A professional clinical nephrology pharmacist, who had 10 years of experience in the management of nephropathy medication, identified DRPs and provided pharmaceutical recommendations to the doctors, nurses and patients. DRPs were identified by this pharmacist by evaluating the appropriateness of drug therapy in terms of indication, dosage, safety, efficacy and cost. Standard guidelines published by authoritative nephrology organization, such as Kidney Disease: Improving Global Outcomes (KDIGO) association, and drug instructions were the main references for determining DRPs. The clinical pharmacist evaluated and addressed DRPs by attending clinical ward rounds with doctors from Monday to Friday, reviewing doctors’ orders daily in the hospital information system (HIS), and providing medication-related education (MRE) for discharged patients from Monday to Friday. At each clinical round, the pharmacist preformed three main tasks: ① Medication reconciliation (MR) aiming to reduce medication discrepancies in the dose or frequency of all medications given within 24 h after admission to the nephrology ward compared with those before admission, in addition to adjustment of anticoagulant or antiplatelet medication for patients requiring renal biopsy. ② Medication evaluation and management (MEM) mainly involving reviews of the route of administration, dosage and incompatibility for new orders by the pharmacist. ③ MRE for discharged patients aiming to improve patient compliance and medical knowledge using educational materials. MRE included best usage of drugs, special drug storage conditions, common adverse effects of drugs, and food and drugs contraindications. When necessary, pharmacist interviewed patients to determine the occurrence of a DRP. The identified DRPs were categorized using PCNE classification tool, version 9.00, and briefly classified by problem (P), cause (C), intervention (I), acceptance (A), and outcome (O) [6].

When a DRP occurred, the clinical pharmacist recorded it, consulted relevant guidelines/drug instructions, and communicated with the clinicians or patients. There were many ways to consult with doctors, including face-to-face discussions, telephone communication, and the use of communication software (WeChat or Ding Talk software). Communication with patients with CKD about DRPs was generally confined to bedside communication. A DRP was considered completely resolved when the clinician followed the pharmacist’s recommendation and made changes to the prescribed medication accordingly before a consequence caused by this DRP during hospitalization. A DRP was considered unresolved if the physician did not follow or implement the pharmacist’s recommendations for prescription modification during hospitalization. If the pharmacist’s recommendation regarding a DRP was not accepted or implemented, the clinical pharmacist recorded any related effects, and carefully monitored changes in laboratory indicators and consequences induced by this DRP during the remainder of the hospital stay. Due to the inclusion of only one clinical pharmacist in this study, all pharmacist interventions and follow-ups were limited to the hospitalization during which the DRP occurred.

### Data collection

All patient data, including sociodemographic characteristics (age, sex, smoking status, and alcohol consumption status), and clinical information (number of complications, number of drugs prescribed, and length of stay), were collected and recorded. All the abovementioned data were queried and extracted from the HIS. The sociodemographic data of all new inpatients with CKD were collected by a graduate student at 9:00 a.m. daily, whereas the clinical data were collected by the pharmacist every week. If certain information was missing in the HIS, the clinical pharmacist inquired about the information.

### Statistical analysis

Categorical variables were expressed as frequencies and percentages, and continuous variables were expressed as means and standard deviations (SDs). The Wilcoxon signed ranks test was used to analyze the changes in DRP quantity before and after the clinical pharmacist’s intervention in CKD patients as the distribution of DRPs in the sample did not conform to a Gaussian distribution. Logistic regression analysis was performed to determine potential determinants of DRPs. The results were reported as odds ratios (ORs) with 95% confidence intervals (CIs). Crude ORs were obtained using logistic regression for a single independent variable. Accordingly, only significant independent variables were analyzed using logistic regression to adjust the OR. The Statistical Package for Social Science (SPSS) (version 26.0) was used for data analysis, and a *p* value < 0.05 was considered statistically significant.

## Results

### Patient characteristics

A total of 914 patients were included, with a mean age of 60.23 ± 17.83 years. 38.29% were men, and 64.44% of patients were diagnosed with stage 5 CKD in this study. The median numbers of comorbid diseases were 3.12 ± 1.31, with hypertension (74.84%) and diabetes mellitus (34.79%) being the most common kinds. The average hospital stay of each patient was nearly 1 week (8.76 ± 4.54 days), and 14.74 ± 8.60 drugs were prescribed (Table 1).

**Table 1:** Demographic and clinical characteristics of the population in the study (N = 914)

Characteristics of CKD patients		N (%)
Age (year), Mean (SD)		60.23(17.83)
Gender		
	Male	350(38.29)
	Female	564(61.71)
Smoker		180(19.69)
Alcohol user		111(12.14)
Stage of CKD patients		
	CKD 1(eGFR ≥ 90mL/min/1.73m^2^)	149(16.30)
	CKD 2(eGFR 60–89 mL/min/1.73m^2^)	84(9.19)
	CKD 3(eGFR 30-59mL/min/1.73m^2^)	56(6.13)
	CKD 4(eGFR 15-29mL/min/1.73m^2^)	36(3.94)
	CKD 5(eGFR <15mL/min/1.73m^2^)	589(64.44)
Common comorbidities		
	Hypertension	684(74.84)
	Diabetes mellitus	318(34.79)
	hyperlipemia	289(31.62)
	Coronary artery disease	122(13.35)
Number of comorbidities, Mean (SD)		3.12(1.31)
Number of prescribed drugs during hospitalization, Mean(SD)		14.74(8.60)
Length of hospital stay (day), Mean(SD)		8.76(4.54)

CKD: chronic kidney disease; eGFR: estimated glomerular filtration rate

### Prevalence of DRPs

During the study period, 463 DRPs were recorded in 420 (45.95%) study participants, and the number of DRPs per patient was 0.51 (SD 0.60). Further analysis showed that 385 patients experienced 1 DRP, 27 patients experienced 2 DRPs, and 8 patients experienced 3 DRPs before the clinical pharmacist’s intervention during hospitalization.

### Classification of DRPs

**Fig. 1:**
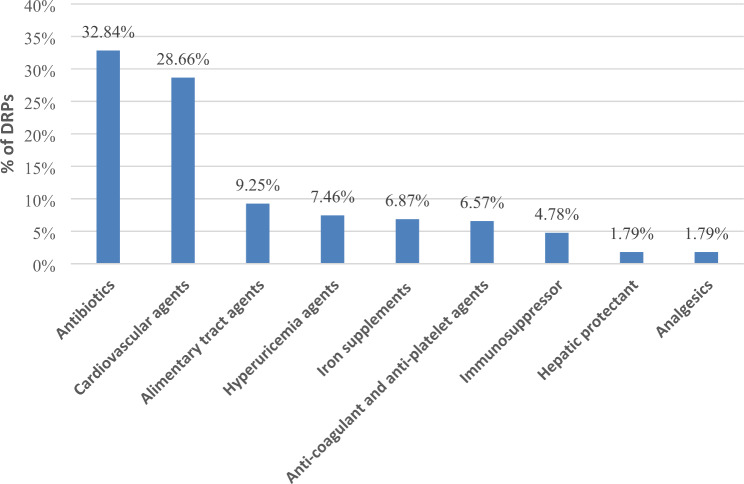
Classifications of drugs involved in DRPs

According to the PCNE classification tool version 9.00, treatment safety accounted for the highest proportion of problems (43.84%), followed by treatment efficacy (43.20%). Drug selection was the most common cause of DRPs (60.26%), followed by dose (29.59%) (Table 2). Unreasonable use of antibiotics and cardiovascular agents were the most common cause of DRPs (32.84% and 28.66%, respectively) (Fig. 1).

**Table 2:** Classification of DRPs identified using PCNE v9.00 (N = 463)

DRPs			Detailed Classification	N (%)
**Problems domain** (total 463)				
	P1		The effectiveness of treatment	200(43.20)
		P1.1	No effect of drug treatment	1(0.50)
		P1.2	Effect of drug treatment not optimal	97(48.50)
		P1.3	Untreated symptoms or indication	102(51.00)
	P2		The safety of treatment	203(43.84)
		P2.1	Adverse drug event (possibly) occurring	203(100.00)
	P3		Other	60(12.96)
		P3.1	Problem with cost-effectiveness of the treatment	20(33.33)
		P3.2	Unnecessary drug-treatment	40(66.67)
**Causes domain** (total 463)				
	C1		Drug selection	279(60.26)
		C1.2	Inappropriate drug (within guidelines but otherwise contraindicated)	88(31.54)
		C1.3	No indication for drug	37(13.26)
		C1.4	Inappropriate combination of drugs, or drugs and herbal medications, or drugs and dietary supplements	16(5.73)
		C1.5	Inappropriate duplication of therapeutic group or active ingredient	9(3.23)
		C1.6	No or incomplete drug treatment in spite of existing indication	119(42.65)
		C1.7	Too many medicines prescribed for indication	10(3.58)
	C2		Drug form	17(3.67)
		C2.1	Inappropriate drug form for this patient	17(100.00)
	C3		Dose selection	137(29.59)
		C3.1	Drug dose too low	24(17.52)
		C3.2	Drug dose too high	83(60.58)
		C3.3	Dosage regimen not frequent enough	6(4.38)
		C3.4	Dosage regimen too frequent	14(10.22)
		C3.5	Dose timing instructions wrong, unclear or missing	10(7.30)
	C4		Treatment duration	5(1.08)
		C4.1	Too short treatment duration	2(40.00)
		C4.2	Too long treatment duration	3(60.00)
	C9		Other	25(5.40)
		C9.1	No or inappropriate monitoring outcome (e.g., TDM)	25(100.00)

PCNE: Pharmaceutical Care Network Europe; DRPs: drug-related problems

### Clinical pharmacist interventions

Approximately half of the clinical pharmacist interventions were provided at the prescriber level, and 49.46% of the interventions were offered at the drug level, and mainly included adjustments of the type and dosage of drugs, as well as stopping or starting some drugs (Table 3). According to the results, 396 (85.53%) pharmaceutical interventions recommendations were accepted, and 67 (14.47%) recommendations were rejected for various reasons (Table 3).

**Table 3:** Clinical pharmacist interventions identified using PCNE v9.00 (N = 463)

DRPs			Detailed Classification	N (%)
**Intervention domain** (total 463)				
	I1		At prescriber level	234(50.54)
		I1.3	Intervention proposed to prescriber	48(20.51)
		I1.4	Intervention discussed with prescriber	186(79.49)
	I3		At drug level	229(49.46)
		I3.1	Drug changed to	45(19.65)
		I3.2	Dosage changed to	57(24.89)
		I3.3	Formulation changed to	7(3.06)
		I3.4	Instructions for use changed to	14(6.11)
		I3.5	Drug paused or stopped	38(16.59)
		I3.6	Drug started	68(29.69)
**Acceptance domain** (total 463)				
	A1		Intervention accepted by patient or prescriber	396(85.53)
		A1.1	Intervention accepted and fully implemented	387(97.73)
		A1.2	Intervention accepted, implemented partially	8(2.02)
		A1.3	Intervention accepted but unimplemented	1(0.25)
	A2		Intervention not accepted	67(14.47)
		A2.1	Intervention unaccepted: not feasible	4(5.97)
		A2.2	Intervention unaccepted: no agreement	61(91.04)
		A2.4	Intervention unaccepted: unknown reason	2(2.99)

PCNE: Pharmaceutical Care Network Europe; DRPs: drug-related problems

### DRP outcomes of pharmacist interventions

In total, 390 DRPs (84.23%) were resolved after implementation of interventions by a clinical pharmacist (Table 4). The proportion of patients who experienced DRPs decreased to 7.77%, with an average of 0.08 (SD 0.28) DRPs during hospitalization after pharmacist intervention. Wilcoxon signed ranks test result showed a significant reduction in the mean number of DRPs after the pharmacist’s intervention in this single center study (p = 0.000).

**Table 4:** Outcome of pharmacist’s intervention with DRPs identified using PCNE v9.00 (N = 463)

DRPs			Detailed Classification	N (%)
**Outcome domain** (total 463)				
	O1		Solved	390(84.23)
		O1.1	DRP totally solved	390(100.00)
	O3		Not solved	73(15.77)
		O3.1	DRP not solved, lack of cooperation of patient	5(6.85)
		O3.2	DRP not solved, lack of cooperation of prescriber	53(72.60)
		O3.3	DRP not solved, intervention not effective	15(20.55)

PCNE: Pharmaceutical Care Network Europe; DRPs: drug-related problems

Seventy-three DRPs (15.77%) occurred even with the intervention of a pharmacist, failure to follow the recommendation by the prescriber was the main reason (72.60%) (Table 4). Lack of cooperation by the patient (6.85%) was another reason. In 15 cases (20.55%), DRP remained unsolved even though the pharmacist’s recommendations were followed by the prescriber (Table 4). Some typical cases are listed in the appendix for ease of understanding.

Among the 56 unresolved DRPs due to lack of cooperation by the patient or prescriber, 10 cases of DRP induced consequences, such as acute kidney disease, mental agitation and elevated blood glucose. The most serious consequence induced by DRP was systemic submucosal hemorrhage in a hemodialysis patient with pneumonia; according to the pharmacist’s evaluation using the World Health Organization (WHO) Uppsala adverse events scale, this symptom was likely caused by an improper dosage of cefoperazone sodium and sulbactam sodium. Among the remaining 46 unsolved DRPs, there was clearly an irrational use of medication, but no severe consequences were reported.

### Contributing factors of DRPs

CKD stage 4, number of comorbid diseases, number of prescribed medications, and hospitalization days were contributing factors of DRPs in both the univariate and multivariate logistic regression models (Table 5). In contrast to the results of the univariate regression analysis, CKD stage 5 showed no statistical significance in the multivariate regression model.

**Table 5:** Determinants of DRPs identified using logistic regression

Determinates	Patients with DRP (total 420), N(%)	Patients without DRP (total 494), N(%)	Univariable logistic regression analysis		Multivariate logistic regression analysis	
			Crude OR (95%CI)	*p* value	Adjusted OR (95%CI)	*p* value
Age (year), Mean(SD)	62.40 ± 17.37	58.36 ± 18.02	1.013 (1.006–1.021)	0.001^*^	1.001(0.993–1.010)	0.771
Gender						
Female	265 (63.10)	299 (60.52)	Reference			
Male	155 (36.90)	195 (39.47)	0.897 (0.686–1.173)	0.426		
Smoking status						
No	331 (78.81)	403 (81.58)	Reference			
Yes	89 (21.19)	91 (18.42)	1.191 (0.859–1.650)	0.294		
Alcohol user						
No	365 (86.90)	438 (88.66)	Reference			
Yes	55 (13.10)	56 (11.34)	1.179 (0.792–1.753)	0.417		
Stage of CKD patients						
CKD 1	48 (11.43)	101 (20.45)	Reference		Reference	
CKD 2	32 (7.62)	52 (10.53)	1.295 (0.741–2.264)	0.365	0.977(0.542–1.761)	0.939
CKD 3	23 (5.48)	33 (6.68)	1.467 (0.778–2.764)	0.236	1.132(0.583–2.198)	0.714
CKD 4	23 (5.48)	13 (2.63)	3.723 (1.738–7.976)	0.001^*^	2.342(1.045–5.253)	0.039^*^
CKD 5	294 (70)	295 (59.72)	2.097 (1.435–3.065)	0.000^*^	1.249(0.809–1.927)	0.315
Number of comorbidities, Mean (SD)	3.34 ± 1.45	2.93 ± 1.15	1.272 (1.148–1.410)	0.000^*^	1.187(1.059–1.329)	0.003^*^
Number of prescribed drugs during hospitalization, Mean(SD)	17.00 ± 9.20	12.86 ± 7.58	1.062 (1.044–1.080)	0.000^*^	1.050(1.031–1.069)	0.000^*^
Length of hospital stay (day), Mean(SD)	9.56 ± 4.46	8.08 ± 4.51	1.076 (1.044–1.108)	0.000^*^	1.035(1.001–1.071)	0.043^*^

DRPs: drug-related problems; OR: odds ratio; CI: confidence interval;^*^ indicates P < 0.05

## Discussion

In this prospective study, we established that the clinical pharmacist’s intervention could identify and address DRPs in hospitalized patients with CKD by attending ward rounds, reviewing order daily and providing MRE. The services of clinical pharmacists have been effective in decreasing DRPs in China [23, 24]. To our knowledge, this is the first large prospective study that was performed for one year to assess the potential role of clinical pharmacist services in hospitalized patients with CKD in China.

According to a systematic review of 20 studies in 2021, the prevalence of DRP in CKD patients ranged from 12% to 87%, with 0.4–1.7 DRPs per patient [14]. In this study, 45.95% (420/914) of CKD inpatients experienced DRPs, with an average of 0.51 (SD, 0.60) DRPs per person before pharmacist intervention. In a 2014 retrospective study conducted in another general hospital in Zhejiang Province, researchers analyzed 1733 lines of medication prescriptions from 202 patients with CKD and found that the prevalence of inappropriate medication prescriptions in hospitalized patients with CKD was 15.18% [9]. These data indicate that DRPs are a common phenomenon in hospitalized patients with CKD in China. The major reason for DRPs was drug/dose selection (89.85%), according to the PCNE classification tool version 9.00, a general DRP analysis method [6]. Regarding drug selection, the irrational use of antibiotics (32.84%) and cardiovascular agents (28.66%) were the main contributors to DRPs, similar to other studies [9, 10, 25]. We found that the most common comorbidities of patients with CKD were hypertension (74.84%) and diabetes mellitus (34.79%), which might explain why the unreasonable use of cardiovascular agents was common. This was largely due to unreasonable drug combinations or inappropriate drug selection in this study. For instance, the combination of a losartan potassium hydrochlorothiazide tablet and irbesartan tablet has been observed in this study. This is a typical irrational drug combination because both drugs contain the same mechanism of action. However, most DRPs due to the wrong dosage were caused by the antimicrobials. This was consistent with the data reported by Yang Ping, with unreasonable dosages of antimicrobial drugs (89.29%) being the most common category [9]. Some antibacterial drugs, such as levofloxacin and vancomycin which are mainly excreted through the kidneys, need dosage adjustment in the patients with end-stage renal disease [12, 13]. Excessive use of these drugs is usually accompanied by the deterioration of renal function. However, dose adjustment was not always strictly implemented, as there were 83 DRPs due to overdose in our study.

In this study, 85.53% of pharmacist’s recommendations were accepted, and 84.23% of DRPs were consequently solved. Through intervention by the pharmacist, the proportion of patients who experienced DRPs decreased from a possible 45.95% to 7.77%, with the average number of DRPs per patient decreasing sharply (possible, 0.51 ± 0.60 vs. actual, 0.08 ± 0.28). These findings suggest that even only one clinical nephrology pharmacist in the nephrology ward, clinical pharmacist can play an important role in facilitating the identification of DRPs in patients with CKD and assisting physicians resolve DRPs in this single center study in China. It should be noted that 67 pharmacist recommendations were not accepted, with objections from doctors being the main reason (91.04%) in this study. Physician’s objections to pharmacist interventions are known to be common [18, 26]. In this hospital the possible reasons for physicians to not accept pharmacist’s interventions might have been: first, when the laboratory indicators in a CKD patient are slightly abnormal, the clinical pharmacist may be more inclined to adjust the medication order. Doctors often tend to pay more attention to dynamic indicator change trends and adjust the prescriptions only when parameters become highly abnormal or relevant clinical symptoms appear. We also noted that in 61 cases of DRPs, the clinicians initially disagreed with the pharmacist, whereas only 53 DRPs were not resolved due to the lack of cooperation by the prescriber (Tables 3 and 4). This indicated that in 8 DRP cases, the clinician eventually adopted the pharmacist’s medication plan. Second, pharmacist prefer medication treatment regimens in line with guidelines or drug instructions; however, physicians consider the efficacy and safety of treatment, patient compliance, financial status of the patient and make clinical decisions after comprehensive consideration. In 5 cases, DRPs remained unsolved due to the lack of cooperation by patients. These findings suggest that pharmacist needs to obtain an in-depth clinical understanding of the needs of patients to provide the most appropriate pharmaceutical advice.

Comorbidities and number of prescribed medicines ≥ 5 are contributing factors in the occurrence of DRPs, and this has been proven in several studies [5, 10, 11]. Two studies have demonstrated that a hospitalization period longer than five days increases the possibility of DRPs [11, 25]. We obtained similar conclusions in this study: the number of comorbid diseases, use of multiple prescription drugs, and an extended length of hospital stay were contributing factors of DRPs. Some studies have shown that CKD stage is an independent risk factor for DRPs [5, 11, 25, 27]. However, we found that only stage 4 CKD was remarkably correlated with DRPs in both the univariate and multivariate logistic regression analyses. The possible reasons are as follows: most patients with stage 5 CKD are receiving regular dialysis, visit the hospital frequently, and receive more attention from clinicians than patients with lower stages. Moreover, doctors clearly know whether a medication is appropriate and how to adjust the dose for dialysis patients. We observed that a tailored approach for patients with stage 4 CKD might be more complicated and difficult for doctors. Dapagliflozin, for instance, is forbidden in patients undergoing dialysis; however, many clinicians ignore the contraindication when the eGFR is < 30 mL/min/1.73 m^2^. CKD stage 5 was a statistically significant variable in the univariate logistic regression model, but significance was not retained in the multiple regression model. This might be due to the high proportion of patients with stage 5 CKD (64.44%) in the total sample. These data remind us that patients with severe renal insufficiency should receive more care from both clinicians and pharmacists, regardless of whether they are undergoing dialysis.

### Limitations

This study has some limitations. First, only one clinical nephrology pharmacist was present in the hospital where the study was conducted. This led to the observed results in this study highly dependent on this pharmacist. If multiple clinical pharmacists together participate in the management of DRPs in hospitalized patients with CKD, the incidence and intervention effect of DRPs might differ from this study’s results. Second, this single-center study is likely to have sampling bias due to the large population base of CKD in China. The data obtained from multi-center study might be closer to the reality.

## Conclusion

This study confirmed that DRPs are common among hospitalized patients with CKD in China. CKD stage 4, comorbidities, use of multiple prescription drugs, and an extended length of hospital stay were contributing factors for DRPs. Even only one clinical nephrology pharmacist in the nephrology ward, clinical pharmacist can play an important role in facilitating the identification of DRPs in patients with CKD and assisting physicians resolve DRPs in this single center study in China.

### Electronic supplementary material

Below is the link to the electronic supplementary material.

Supplementary Material 1

## Data Availability

All data generated during this study are included in this published article. The dataset analyzed during the study is available from the corresponding author upon reasonable request.

## Abbreviations

CI: Confidence interval
CKD: Chronic kidney disease
CK-NET: China Kidney Disease Network
DRP: Drug-related problem
eGFR: Estimated glomerular filtration rate
HIS: Hospital information system
KDIGO: Kidney Disease: Improving Global Outcomes
MEM: Medication evaluation and management
MR: Medication reconciliation
MRE: Medication-related education
OR: Odds ratio
PCNE: Pharmaceutical Care Network Europe
SD: Standard deviation
