# Supplementary material for: Drug-related problems in hospitalized patients with chronic kidney diseases and clinical pharmacist interventions
Source: BMC Geriatr. 2023 Dec 13;23:849. doi: 10.1186/s12877-023-04557-y (PMC10717358; doi:10.1186/s12877-023-04557-y)
Supplement: Supplementary file 1 — Supplementary Material 1 [file 12877_2023_4557_MOESM1_ESM.docx]

**Table 1** Some typical DRP cases identified using PCNE v9.00.

| DRP case | Causes (C) | Description and pharmacist’s recommendations | Acceptance (A) | Outcome(O) | Reasons for clinical pharmacist’s intervention failure or unsolved DRP |
| --- | --- | --- | --- | --- | --- |
| Case 1: A 58-year-old patient with CKD stage 4 was treated with cefotaxime sodium (2 g intravenous infusion (ivgtt) for 8h) for nephropyelitis. | C3.2 Drug dose too high | According to instructions, cefotaxime sodium maintenance should be halved due to severe renal dysfunction. | A 1.1 Intervention accepted and fully implemented by the prescriber. | O 1.1 DRP totally solved.  No ADE occurred. | _ |
| Case 2: A 62-year-old patient with uremia and kidney cancer who developed very severe nausea and vomiting during oxycodone use. | C1.2 Inappropriate drug use | The pharmacist recommended using fentanyl transdermal patches instead of oxycodone. This might reduce the effects of oral drugs on the gastrointestinal tract. | A 1.1 Intervention accepted and fully implemented by the prescriber. | O 3.3 DRP not solved; intervention not effective.  The gastrointestinal symptoms in this case were relieved but did not disappear. | Nausea and vomiting in this patient might be related to the pharmacological mechanisms of opioid receptor antagonists. However, opioid receptor antagonists could not be discontinued. After the doctor prescribed antiemetics for the patient on the advice of the pharmacist, the gastrointestinal symptoms were further relieved. |
| Case 3: A 54-year-old patient with lupus nephritis and hypertension was regularly treated with losartan potassium. An electrolyte test during hospitalization revealed slightly elevated potassium concentration (5.36 mmol/L). | C1.2 Inappropriate drug use | Losartan potassium can cause an increase in blood potassium concentration, which had already been high in this case. Hydrochlorothiazide was recommended to be added, and electrolyte levels were recommended to be tested regularly. | A 1.2 Intervention implemented partially by the prescriber. | O 1.1 DRP totally solved.  Blood potassium level returned to normal three days later.  No ADE occurred. | The clinician believed that blood potassium was only slightly elevated; therefore, hydrochlorothiazide was not required to be temporarily added. However, the prescriber took the advice of checking electrolyte levels regularly. |
| Case 4: A 63-year-old patient with nephrotic syndrome received a combination of clonazepam and alprazolam daily for insomnia. | C1.4 Inappropriate combination of drugs, or drugs and herbal medications, or drugs and dietary supplements | Clonazepam and alprazolam are both long-acting sedative hypnotics.  The incidence of adverse reactions, including oversedation, could increase when both drugs were used together. The clinical pharmacist suggested that it might be appropriate to only use one kind of a long-acting insomnia drug. | A 1.3 Intervention accepted but not implemented. | O 3.1 DRP not solved owing to the lack of patient cooperation.  The patient occasionally suffered from daytime burnout. However, this symptom did not affect the patient's normal life. | The doctor adopted the pharmacist's advice. However, this patient with intractable insomnia, had been treated for insomnia with two drugs together for several years. The discontinuation of either insomnia medication was resolutely denied by the patient. |
| Case 5: A 69-year-old man on hemodialysis was treated with voriconazole (120 mg ivgtt for 12h) for fungal infection. | C9.1 No monitoring outcome (e.g. TDM) | Voriconazole can be cleared by hemodialysis. It was recommended to test the blood concentration of voriconazole. | A 2.1 intervention not accepted as it was not feasible. | O 3.2 DRP not solved owing to the lack of prescriber cooperation.  The patient was cured of fungal infection one week later.  No ADE occurred. | Voriconazole blood concentration detection had not been conducted in 2020. The doctor refused to send the blood sample to other testing companies. |
| Case 6: A 59-year-old patient with diabetic nephropathy was treated with mixed protamine zinc recombinant human insulin injection 70/30 (Humulin 70/30; 18 IU ih for 12h). The patient had a slightly elevated 2-hour postprandial blood sugar in the midnoon. | C1.2 Inappropriate drug use | To control blood glucose smoothly, compared with a premixed insulin (every 12 h) regimen, a short-acting insulin (before meals) combined with long-acting insulin (at night) regimen was a better medication choice. | A 2.2 Intervention was not accepted as it was not agreed upon by the patient. | O 3.2 DRP not solved owing to the lack of prescriber cooperation.  The patient still experienced occasional fluctuations in blood glucose. | The doctor agreed with the pharmacist’s suggestion and was very aware of the patient's medication compliance. This patient used to reduce the number of insulin injections arbitrarily owing to discomfort after a high number of insulin injections. Clinicians used the premixed insulin regimen to improve patient compliance. |
| Case 7: A 75-year-old woman with lupus nephritis had frequent fevers and urinary tract infection. | C1.6 No or incomplete drug treatment despite existing indication | The pharmacist advised the doctor to use thymulin to boost the patient’s immunity. | A 2.4 Intervention unaccepted: unknown reason | O 3.1 DRP not solved owing to the lack of patient cooperation.  The patient received adequate antibiotics and was discharged.  No ADE occurred. | The doctor followed the pharmacist's advice and prescribed thymulin. Whereas, the patient refused the prescription and rejected to give a reason. Rejection was probably due to the patient being uninsured and thymulin being expensive. |

PCNE: Pharmaceutical Care Network Europe; DRP: drug-related problem; ivgtt: intravenous infusion; ih: subcutaneous injection
